# Association between interleg systolic blood pressure difference and apparent peripheral neuropathy in US adults with diabetes: a cross-sectional study

**DOI:** 10.1186/s41043-023-00475-2

**Published:** 2023-11-24

**Authors:** Xipeng Lin, Zhihao Liu, Haoyu Weng, Xu Liu, Shengcong Liu, Jianping Li

**Affiliations:** 1 Department of Cardiology, Peking University First Hospital, Beijing, 100083 People’s Republic of China; 2 Key Laboratory of Molecular Cardiovascular Sciences of Ministry of Education, Health Science Center, Peking University, Beijing, People’s Republic of China

**Keywords:** Age, Diabetes, Interleg systolic blood pressure difference, Apparent peripheral neuropathy, Semmes–Weinstein monofilament test

## Abstract

**Background:**

Interleg systolic blood pressure difference (ILSBPD) is associated with peripheral artery disease, but the relationship between ILSBPD and apparent peripheral neuropathy in diabetic patients remains unclear. We explored the relationship between ILSBPD and apparent peripheral neuropathy and examined the possible effect modifiers in US adults with diabetes.

**Methods:**

One thousand and fifty-one diabetic participants were included in the study with complete data on systolic blood pressure of the lower extremities and Semmes–Weinstein 10-g monofilament testing from the 1999–2004 National Health and Nutritional Examination Surveys. Systolic blood pressure in the lower extremities was measured using an oscillometric blood pressure device with the patient in the supine position. Apparent peripheral neuropathy was defined as the presence of monofilament insensitivity.

**Results:**

Every 5-mmHg increment in ILSBPD is associated with an about 14% increased risk of apparent peripheral neuropathy in crude model, but after adjustment for covariates, the correlation became nonsignificant (*P* = 0.160). When participants were divided into groups based on ILSBPD cutoffs of 5, 10 and 15 mmHg in different analyses, there was a significantly increased risk of apparent peripheral neuropathy in the ILSBPD ≥ 15 mmHg group (OR 1.79, 95% CI 1.11–2.91, *P* = 0.018), even after adjusting for confounders. In subgroup analysis, no interaction effect was found (all *P* for interaction > 0.05).

**Conclusions:**

In US adults with diabetes, an increase in the ILSBPD (≥ 15 mmHg) was associated with a higher risk of apparent peripheral neuropathy.

## Background

Peripheral neuropathy covers a variety of clinicopathologies that may be associated with dysfunction of the peripheral nervous system [1]. Diabetes mellitus is the most common cause of peripheral neuropathy in Western societies, with a prevalence of up to 30–66% of diabetic patients [2]. In addition to the production of advanced glycation endproducts, reactive oxygen species and inflammatory factors caused by chronic hyperglycemia, structural microvasculature damage is an important cause of nerve dysfunction, as neuropathy is essentially a microvascular disorder [3–8]. Participants with peripheral neuropathy often experience progressive neuropathic pain and decreased sensation, which lead to skin breakdown, infection, ulceration and eventually non-traumatic amputations, with associated depressive symptoms and poor quality of life [9–12]. Due to the insidious onset of peripheral neuropathy and heterogeneous clinical manifestations, the prevalence of peripheral neuropathy in diabetic participants is severely underestimated. The Semmes–Weinstein monofilament test is an inexpensive, noninvasive and easy-to-administer test used for screening apparent peripheral neuropathy. It is a rapid test with high specificity but lower sensitivity [13]. Due to its low cost and ease of application, it is widely used in both clinical and self-assessment settings.

Interleg systolic blood pressure difference (ILSBPD) refers to the absolute systolic blood pressure (SBP) difference between bilateral lower extremities. The underlying mechanism of a moderate to severe ILSBPD is that the arterial stenosis induced by plaque, aneurysm, or arteritis is not symmetrically distributed between the limbs [14, 15]. As the lower extremities are more prone to peripheral artery disease (PAD) than the upper extremities, ILSBPD is probably a better predictor of PAD than the interarm SBP difference [16, 17]. In addition, accumulating data indicate that ILSBPD is associated with diabetic nephropathy [18], cardiovascular risk factors [19, 20], left ventricular mass index [21], prevalent stroke [22], and cardiovascular and overall mortality [23, 24], which further highlights its clinical importance.

Previous studies have established a correlation between peripheral neuropathy and PAD evaluated by the ankle-brachial index (ABI) and toe-brachial index (TBI) [25, 26], and between peripheral neuropathy and clinical macroangiopathy or a history of intermittent claudication [27]. However, these small-scale studies have not thoroughly examined any possible effect modifiers, and to our knowledge, no study has evaluated the relationship between ILSBPD and apparent peripheral neuropathy.

The current study aimed to investigate the relationship between ILSBPD and apparent peripheral neuropathy, and to examine the possible effect modifiers in US adult diabetic participants using data from the 1999–2004 National Health and Nutritional Examination Surveys (NHANES).

## Methods

### Study population

All participants were from the NHANES. The NHANES is a large-scale, multicenter, cross-sectional, ongoing survey of a non-institutionalized US population conducted by the National Center for Health Statistics; the detailed methodology is described on the official website [28]. Briefly, individuals aged 60 years and older, African-Americans and Hispanics were over-sampled to produce more reliable statistics. The three main components of the study included an interview in each participant’s home, a physical examination, and several medical and laboratory tests. Comorbidities were self-reported. The National Center for Health Statistics Ethics Review Board approved the protocols, and informed consent was obtained from all subjects.

**Fig. 1:**
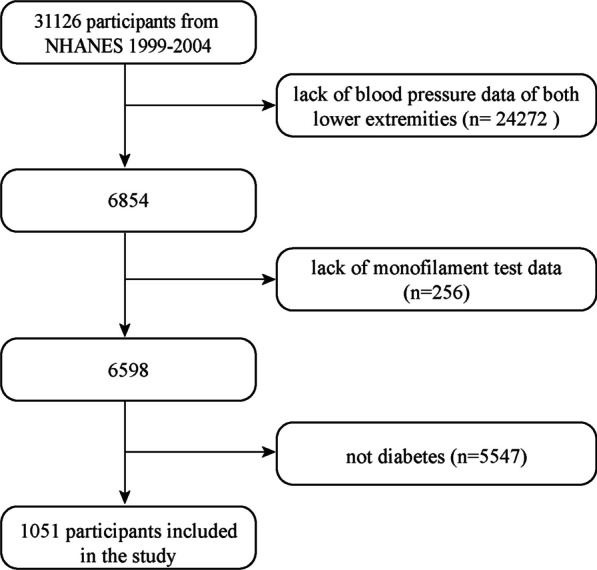
Flowchart of the study

We performed a secondary analysis of data from three 2-year NHANES cycles: 1999–2000, 2001–2002 and 2003–2004; the total number of participants was 31 126. The exclusion criteria of the current study were: (1) lack of complete data on blood pressure of the lower extremities (*n *= 24 272); (2) lack of monofilament test data (*n *= 256); and (3) non-diabetic participants who had no history of diabetes, a fasting glucose of < 7 mmol/L and a glycosylated hemoglobin (HbA1c) of ≤ 6.5% (*n *= 5547). A final total of 1051 diabetic participants were included in the analysis (Fig. 1).

### Variables

#### Dependent variable

The outcome of the analysis was the prevalence of apparent peripheral neuropathy, which was assessed by the Semmes–Weinstein monofilament test. The examiner used a standard monofilament to apply 10 g of force to three different sites on the bottom of each foot. Participants were asked to respond when they felt the pressure. A sensate site was defined as a site at which the first response was correct or at which two of three tests were correct. An insensate site was defined as a site at which at least two of three tests were incorrect or the patient was ‘unable to determine’ the pressure. Apparent peripheral neuropathy was defined as the presence of one or more insensate sites on either foot [29–31].

#### Independent variable

The independent variable of the study was the ILSBPD. With the patient in the supine position, blood pressure was measured using a vascular testing device (Parks Mini-Lab IV, Model 3100; Aloha, OR) in the right arm and both ankles. Blood pressure was measured twice in subjects aged 40–59 years, but only once in subjects aged 60 years and older [32]. ILSBPD refers to the absolute value of the SBP difference between the lower extremities.

#### Covariates

Continuous variables consisted of age (years), duration of diabetes (years), brachial SBP (mmHg), total cholesterol (TCHO, mmol/L), low-density lipoprotein-cholesterol (LDL-c, mmol/L), high-density lipoprotein-cholesterol (HDL-c, mmol/L) and HbA1c (%).

Categorical variables included gender (male, female), race/ethnicity (Hispanic, non-Hispanic white, non-Hispanic black and other race), peripheral artery disease status (yes, no), history of hypertension (yes, no) and history of hypercholesterolemia (yes, no).

Demographic data including age, gender, race, history of hypertension and history of hypercholesterolemia were self-reported by the participants. Blood samples were collected and stored under appropriate temperature conditions until they were shipped to Johns Hopkins University Lipoprotein Laboratory for testing.

#### Statistical analysis

Analyses were performed using EmpowerStats (http://www.empowerstats.com) and the statistical package R (3.2.3 version). The characteristics of the subjects were stratified by ILSBPD. Continuous variables with normal distribution were expressed as means with standard deviations (SD), and the Student’s t-test was used to assess the difference between two groups. Categorical variables were presented as numbers and percentages, and the Chi-square test was used to assess the difference between groups. An adjusted smoothing spline plot of ILSBPD and apparent peripheral neuropathy was analyzed using a multivariate logistic regression model. In three separate analyses, participants with ILSBPD were then divided into two groups based on cutoff values of 5 mmHg, 10 mmHg and 15 mmHg. A multivariable logistic regression model was used to analyze the relationship between each ILSBPD group and apparent peripheral neuropathy. Possible modifications of the association between ILSBPD and apparent peripheral neuropathy were further investigated by stratified analyses. Interactions were examined by including interaction terms in the regression models. A two-sided *P* value of < 0.05 was considered significant.

## Results

### Characteristics of the study participants

The study comprised 1051 diabetic participants. The mean (SD) age was 62.5 (11.2) years, and 55.9% were male. The demographics and clinical characteristics of the study participants stratified by ILSBPD are listed in Table 1. The mean (SD) age of the ILSBPD < 10 mmHg and ILSBPD ≥ 10 mmHg groups was 61.3 (11.1) years and 65.1 (10.8) years, respectively. The ILSBPD ≥ 10 mmHg group comprised 336 (32.0%) participants; compared with the other ILSBPD group, the ILSBPD ≥ 10 mmHg group was significantly older, had a higher prevalence of apparent peripheral neuropathy and had a higher brachial SBP. There were no significant differences between the three ILSBPD groups regarding sex composition, race composition, prevalence of comorbidities (including history of hypertension and hypercholesterolemia), body mass index, TCHO, LDL-c, HDL-c, fasting glucose level and HbA1c level.

**Table 1:** Characteristics of study participants

Variables	Total (*N *= 1051)	ILSBPD < 10 mmHg (*N *= 715)	ILSBPD ≥ 10 mmHg (*N *= 336)	*P* value
Male, *N* (%)	587 (55.9)	393 (55.0)	194 (57.7)	0.398
Age, year	62.5 (11.2)	61.3 (11.1)	65.1 (10.8)	< 0.001
Race, *N* (%)				0.287
Hispanic	338 (32.2)	235 (32.9)	103 (30.7)	
Non-Hispanic white	433 (41.2)	283 (39.6)	150 (44.6)	
Non-Hispanic black	239 (22.7)	165 (23.1)	74 (22.0)	
Others	41 (3.9)	32 (4.5)	9 (2.7)	
Body measures				
BMI, kg/m^2^	30.7 (5.8)	30.8 (6.0)	30.4 (5.5)	0.232
Brachial SBP, mmHg	134.1 (20.3)	132.2 (19.3)	138.1 (21.7)	< 0.001
Peripheral neuropathy prevalence, *N* (%)	243 (23.1)	143 (20.0%)	100 (29.8)	< 0.001
Peripheral artery disease, *N* (%)	117 (11.2)	30 (4.2)	87 (26.1)	< 0.001
Medical history, *N* (%)				
Hypertension history	656 (62.4)	429 (60.0)	227 (67.6)	0.060
Hypercholesterolemia history	508 (54.6)	349 (55.2)	159 (53.4)	0.841
Laboratory test				
TCHO, mmol/L	5.3 (1.3)	5.3 (1.3)	5.2 (1.2)	0.311
LDL-c, mmol/L	3.0 (0.9)	3.1 (0.9)	2.9 (0.9)	0.238
HDL-c, mmol/L	1.2 (0.4)	1.2 (0.4)	1.2 (0.4)	0.893
Fasting glucose, mmol/L	9.1 (3.8)	9.1 (3.6)	9.2 (4.1)	0.735
HbA1c, %	7.5 (1.8)	7.5 (1.9)	7.3 (1.8)	0.111

Variables are presented as mean (SD) or *N* (%)

*BMI* Body mass index, *SBP* systolic blood pressure, *TCHO* total cholesterol, *LDL-c* low-density lipoprotein-cholesterol, *HDL-c* high-density lipoprotein-cholesterol, *HbA1c* glycosylated hemoglobin

### Association between ILSBPD and apparent peripheral neuropathy

**Fig. 2:**
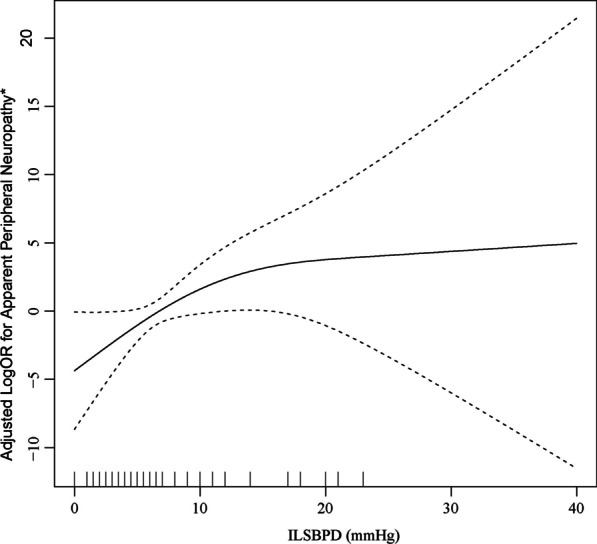
Association between interleg systolic blood pressure difference (ILSBPD) and the risk of apparent peripheral neuropathy. *Adjusted for age, gender, race, brachial systolic blood pressure (SBP), glycosylated hemoglobin (HbA1c), total cholesterol (TCHO), high-density lipoprotein-cholesterol (HDL-c), peripheral artery disease status, duration of diabetes, history of hypertension and hypercholesterolemia

The smoothing spline plot in Fig. 2 indicates a positive relationship between ILSBPD and the risk of apparent peripheral neuropathy. Table 2 shows that continuous ILSBPD was positively correlated with the prevalence of apparent peripheral neuropathy in crude model and adjusted model I (age, race and gender for adjustment). However, after adjusting for all confounding factors in adjusted model II, including age, gender, race, brachial SBP, history of hypertension, history of hypercholesterolemia, peripheral artery disease status, duration of diabetes, HbA1c, TCHO and HDL-c levels, the correlation became not significant (*P *= 0.160). When participants were divided into three groups based on ILSBPD cutoff values of 5 mmHg, 10 mmHg and 15 mmHg, with the lower category as the reference, the ORs significantly increased as the cutoff value increased in a crude model (OR 1.41, 95% CI 1.05–1.91 in the ILSBPD ≥ 5 mmHg group; OR 1.69, 95% CI 1.26–2.28 in the ILSBPD ≥ 10 mmHg group; OR 2.26, 95% CI 1.62–3.15 in the ILSBPD ≥ 15 mmHg group). Adjusted model I was adjusted, and the risk of apparent peripheral neuropathy was significantly increased in the ILSBPD ≥ 10 mmHg group (OR 1.51, 95% CI 1.11–2.06) and ILSBPD ≥ 15 mmHg group (OR 2.07, 95% CI 1.47–2.93). Adjusted model II was adjusted for all covariables; the results in ILSBPD ≥ 15 mmHg group were similar to those in adjusted model I (OR 1.79, 95% CI 1.11–2.91). However, ILSBPD ≥ 10 mmHg was not significantly associated with apparent peripheral neuropathy in adjusted model II (OR 1.43, 95% CI 0.94–2.16).

**Table 2:** Multiple analysis of ILSBPD and peripheral neuropathy

ILSBPD, mmHg	*N*	Peripheral neuropathy presence, *N* (%)	Crude model		Adjusted model I		Adjusted model II	
			OR (95% CI)	*P* value	OR (95% CI)	*p* value	OR (95% CI)	*P* value
Continuous*	1051	243 (23.1)	1.14 (1.07, 1.21)	< 0.001	1.11 (1.04, 1.19)	0.002	1.07 (0.98, 1.16)	0.160
*Group 1*								
< 5	411	80 (19.5)	*Ref.*		*Ref.*		*Ref.*	
≥ 5	640	163 (25.5)	1.41 (1.05, 1.91)	0.025	1.23 (0.90, 1.68)	0.192	1.08 (0.71, 1.63)	0.733
*Group 2*								
< 10	715	143 (20.0)	*Ref.*		*Ref.*		*Ref.*	
≥ 10	336	100 (29.8)	1.69 (1.26, 2.28)	0.001	1.51 (1.11, 2.06)	0.008	1.43 (0.94, 2.16)	0.094
*Group 3*								
< 15	852	171 (20.1)	*Ref.*		*Ref.*		*Ref.*	
≥ 15	199	72 (36.2)	2.26 (1.62, 3.15)	< 0.001	2.07 (1.47, 2.93)	< 0.001	1.79 (1.11, 2.91)	0.018

*Model I* Adjusted for age, gender and race

*Model II* Adjusted for age, gender, race, brachial SBP, HbA1c, TCHO, HDL-c, hypertension history, hypercholesterolemia history, peripheral artery disease status and duration of diabetes

ILSBPD interleg systolic blood pressure difference, SBP systolic blood pressure, HbA1c glycosylated hemoglobin, TCHO total cholesterol, HDL-c high-density lipoprotein-cholesterol

*Every 5-mmHg increment of ILSBPD

### Assessment of interaction

**Fig. 3:**
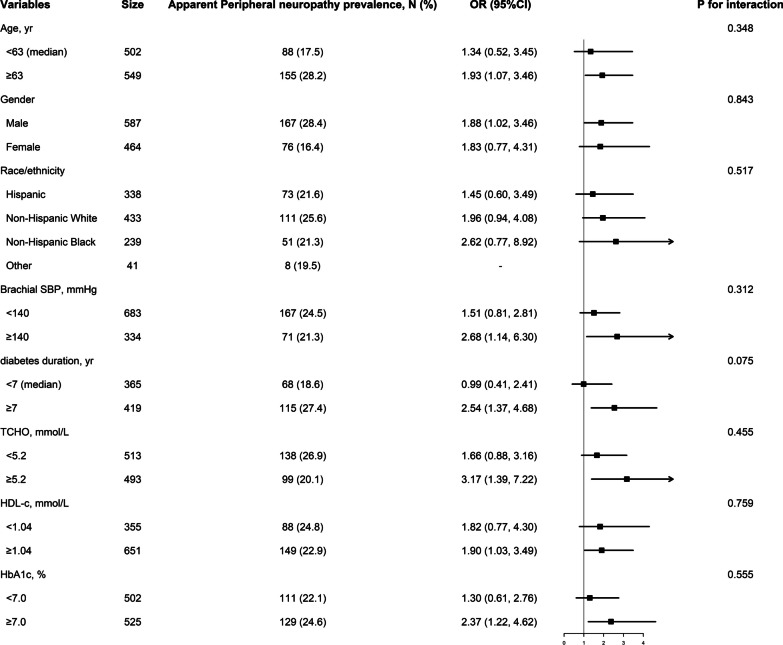
Forrest plots of the association between ILSBPD ≥ 15 mmHg and apparent peripheral neuropathy in various subgroups. Adjusted, if not stratified, for age, gender, race, brachial systolic blood pressure (SBP), glycosylated hemoglobin (HbA1c), total cholesterol (TCHO), high-density lipoprotein-cholesterol (HDL-c), peripheral artery disease status, duration of diabetes, history of hypertension and hypercholesterolemia. ILSBPD stands for interleg systolic blood pressure difference

The previous analysis revealed that an ILSBPD of ≥ 15 mmHg was an independent risk factor for apparent peripheral neuropathy. In order to investigate whether there was any interaction between these two factors, we conducted further subgroup analysis. Figure 3 demonstrates that no significant modification effect was observed in any of the subgroups (all *P* for interaction > 0.05), which supports the robust correlation between an ILSBPD of ≥ 15 mmHg and apparent peripheral neuropathy.

## Discussion

In this population-based, cross-sectional study, we investigated the association between ILSBPD and apparent peripheral neuropathy in US adults with diabetes. To our knowledge, this is the first study to reveal a positive association between an increased ILSBPD and risk of apparent peripheral neuropathy. Furthermore, the subgroup analysis indicated that there was no interaction between the correlation of the two variables.

Previous studies have investigated the association between PAD and peripheral neuropathy in diabetic participants. A cross-sectional study of 775 participants showed that an abnormal ABI (< 0.8) is associated with a 44% increased risk of neuropathy [26]. A Swedish study of 156 diabetic participants revealed that peripheral sensory neuropathy is an independent risk factor for PAD defined as clinical macroangiopathy (no pulses) or a history of intermittent claudication [27]. Another small-scale study discovered that an ABI < 0.9 has a specificity of 90.7% for detecting neuropathy [33]. In addition, TBI is also significantly associated with peripheral neuropathy [25].

Previous studies have confirmed the bidirectional association between PAD and peripheral neuropathy. However, the sample size in these studies has been small and PAD has been assessed on the basis of the ABI, TBI, or clinical characteristics. Our study adopted the ILSBPD, a relatively new variable and added new evidence for the association between PAD and peripheral neuropathy. Furthermore, the comparatively larger sample size in the present study might have provided more power to disclose the associations and enable the exploration of effect modifiers.

The main finding of our study was the positive association between ILSBPD and apparent peripheral neuropathy in adults with diabetes. Although the correlation was not dose-dependent, having an ILSBPD of ≥ 15 mmHg was found to be independently associated with apparent peripheral neuropathy.

In contrast to the interarm SBP difference, for which guidelines have proposed a normal value of < 15 mmHg [34, 35], there is no consensus on the threshold value for ILSBPD. Some studies have proposed that an ILSBPD of ≥ 15 mmHg may be the cutoff point for PAD diagnosis, mortality prediction and the detection of albuminuria and abnormal cardiovascular functional variables [18, 19, 24, 36, 37]. However, an ILSBPD of ≥ 10 mmHg also shows acceptable accuracy and high specificity for PAD diagnosis [38, 39]. Based on our findings, it appears that an ILSBPD ≥ 15 mmHg could be considered as a suitable threshold for detecting apparent peripheral neuropathy, given that an ILSBPD of ≥ 15 mmHg was identified as an independent risk factor for the condition, as opposed to lower cutoff values such as ILSBPD of ≥ 5 mmHg or ILSBPD of ≥ 10 mmHg. One possible reason for the inconsistency among studies of ILSBPD threshold values is the variations in clinical characteristics and cardiovascular conditions among study populations, such as the inclusion of a community-based population and/or participants with diabetes or hemodialysis [22]. Determining the cutoff point for detecting peripheral neuropathy by ILSBPD has significant clinical value. Patients can obtain the data of ILSBPD during the blood pressure measurement. If this value is greater than or equal to 15 mmHg, the patient's risk of developing peripheral neuropathy is significantly increased, and they need further medical evaluation to assess whether peripheral neuropathy is present.

Although ILSBPD and ABI or TBI do not have the same clinical significance, the mechanisms of the association between ILSBPD and peripheral neuropathy might be similar to that between ABI or TBI and peripheral neuropathy, as there was an overlap between individuals with increased ILSBPD and those with an abnormal ABI or TBI. From a pathophysiological point of view, an increased ILSBPD represents low blood flow in at least one lower extremity. Evidence from human and animal studies has shown that reduced nerve blood flow and increased endoneurial vascular resistance can lead to endoneurial hypoxia, which damages the cell bodies in dorsal root ganglia and axons in nerves, causing peripheral neuropathy [40, 41].

The current study has several limitations. First, it was a cross-sectional study without follow-up data; therefore, the causal relationship between ILSBPD and apparent peripheral neuropathy cannot be clarified. Second, the presence of apparent peripheral neuropathy was evaluated by the Semmes–Weinstein 10-g monofilament test, which is commonly used as a screening tool for apparent peripheral neuropathy but is likely to be interfered with by subjective factors, such as patient inattention and thickening of the skin. Additionally, as demographic data were self-reported by participants, there may be potential memory bias. Third, the NHANES excluded subjects deemed unsuitable for the examination of lower-extremity disease, such as participants with bilateral amputations, lesions and severe obesity, who are indeed at high risk of lower-extremity disease. Therefore, the actual prevalence of lower-extremity disease is underestimated, which might have influenced our conclusion.

## Conclusions

In a population of US adults with diabetes, an increase in ILSBPD (≥ 15 mmHg) was associated with higher risk of apparent peripheral neuropathy.

## Data Availability

The datasets analyzed in the current study are available in the National Health and Nutritional Examination Surveys repository, https://wwwn.cdc.gov/nchs/nhanes/Default.aspx.

## Abbreviations

ILSBPD: Interleg systolic blood pressure difference
PAD: Peripheral artery disease
OR: Odds ratio
CI: Confidence interval
SBP: Systolic blood pressure
ABI: Ankle-brachial index
TBI: Toe-brachial index
NHANES: National Health and Nutritional Examination Surveys
HbA1c: Glycosylated hemoglobin
SD: Standard deviations
